# Cross-Species Analysis of Innate Immune Antagonism by Cytomegalovirus IE1 Protein

**DOI:** 10.3390/v14081626

**Published:** 2022-07-26

**Authors:** Franziska Rothemund, Myriam Scherer, Eva-Maria Schilling, Johannes Schweininger, Yves A. Muller, Thomas Stamminger

**Affiliations:** 1 Institute of Virology, Ulm University Medical Center, 89081 Ulm, Germany; franziska.rothemund@uni-ulm.de (F.R.); myriam.scherer@uni-ulm.de (M.S.); eva-maria.schilling@uni-ulm.de (E.-M.S.); 2 Division of Biotechnology, Department of Biology, Friedrich-Alexander-University Erlangen-Nürnberg, 91052 Erlangen, Germany; johannes.schweininger@fau.de (J.S.); yves.muller@fau.de (Y.A.M.)

**Keywords:** cytomegalovirus, HCMV, RCMV, immediate early, innate immunity, IE1, PML, FEN1, STAT2

## Abstract

The human cytomegalovirus (CMV) immediate early 1 (IE1) protein has evolved as a multifunctional antagonist of intrinsic and innate immune mechanisms. In addition, this protein serves as a transactivator and potential genome maintenance protein. Recently, the crystal structures of the human and rat CMV IE1 (hIE1, rIE1) core domain were solved. Despite low sequence identity, the respective structures display a highly similar, all alpha-helical fold with distinct variations. To elucidate which activities of IE1 are either species-specific or conserved, this study aimed at a comparative analysis of hIE1 and rIE1 functions. To facilitate the quantitative evaluation of interactions between IE1 and cellular proteins, a sensitive NanoBRET assay was established. This confirmed the species-specific interaction of IE1 with the cellular restriction factor promyelocytic leukemia protein (PML) and with the DNA replication factor flap endonuclease 1 (FEN1). To characterize the respective binding surfaces, helix exchange mutants were generated by swapping hIE1 helices with the corresponding rIE1 helices. Interestingly, while all mutants were defective for PML binding, loss of FEN1 interaction was confined to the exchange of helices 1 and 2, suggesting that FEN1 binds to the stalk region of IE1. Furthermore, our data reveal that both hIE1 and rIE1 antagonize human STAT2; however, distinct regions of the respective viral proteins mediated the interaction. Finally, while PML, FEN1, and STAT2 binding were conserved between primate and rodent proteins, we detected that rIE1 lacks a chromatin tethering function suggesting that this activity is dispensable for rat CMV. In conclusion, our study revealed conserved and distinct functions of primate and rodent IE1 proteins, further supporting the concept that IE1 proteins underwent a narrow co-evolution with their respective hosts to maximize their efficacy in antagonizing innate immune mechanisms and supporting viral replication.

## 1. Introduction

Human betaherpesvirus 5, also called human cytomegalovirus (HCMV), is a ubiquitous pathogen that rarely causes symptoms in healthy individuals but can lead to severe or even life-threatening diseases in immunocompromised patients and newborns (reviewed in [1]). Characteristic of betaherpesviruses, HCMV has a slow replication cycle and shows a high level of host specificity since it cannot productively replicate in cells of other species [2].

During lytic HCMV replication, viral proteins are expressed in three distinct phases termed immediate early (IE), early (E), and late (L) phase [3]. The immediate early 1 (IE1) protein, expressed very early after infection, is important for initiating the lytic replication cycle, especially under low-multiplicity conditions [4]. HCMV IE1 (hIE1, 491 amino acids (aa) is a multifunctional protein. Its structure can be subdivided into a short N-terminal domain harbouring a nuclear localisation sequence (NLS, aa 1-24), a core domain (aa 24-382), an acidic region (aa 373-475), and a chromatin tethering domain (CTD, aa 467-491) [5,6,7,8]. The NLS domain is required for nuclear transport. It has previously been demonstrated that the large IE1 core is essential to antagonizing promyelocytic leukemia nuclear bodies (PML-NBs), which are part of the intrinsic immune system [7]. These multiprotein complexes appear as dot-like structures of the cell nucleus and can induce the silencing of viral gene expression. PML-NBs are formed by the main organiser promyelocytic leukemia (PML) protein and further restriction factors like ATRX, Sp100, and hDaxx [9,10,11]. IE1 has been shown to inhibit the de novo SUMOylation of PML, followed by PML-NB disruption and inactivation of the intrinsic immune barrier [12,13].

Scherer et al. solved the structure of the rhesus CMV IE1 core, which revealed an unusual, all alpha-helical, femur-like fold [7]. This structure of IE1 is conserved in different species: the core domains of rat CMV IE1 (rIE1) and rhesus CMV IE1 (rhesIE1) show high structural similarity to human IE1 (hIE1) core apart from small differences. All proteins contain a stalk between a C- and N-terminal head region. While the hIE1 core is built by eleven helices, the rIE1 core (aa 30-392) is formed by twelve. Furthermore, helix 2 of the rIE1 core is displaced, and helix 1 is shorter than in the hIE1 core. Interestingly, the folding is conserved, although the sequence identity between primate and rodent IE1 is very low [14]. The core domains of hIE1 and rIE1 bind the natural host PML-NBs via coiled-coil interactions. Despite the structural similarity, this interaction and deSUMOylation and disruption of PML-NBs are species-dependent activities and do not occur during cross-species infection. These findings show that PML NBs contribute to a species barrier against CMVs [7,14].

In addition to PML antagonization, hIE1 plays a major role in the transactivation of viral promoters, as it enhances the transactivation capacity of IE2 [15,16]. It was shown that a possible transactivation site of IE1 is located in the core domain [16]. While Sandford et al. observed that rat CMV IE2 (rIE2) can transactivate heterologous promotors, the role of rIE1 in transactivation has not been demonstrated yet [17]. Furthermore, hIE1 also influences other cellular mechanisms in a pro-viral manner. Schilling et al. detected that the hIE1 core interacts with the flap endonuclease one protein (FEN1) that plays a role in the DNA damage response pathway. Due to this interaction, hIE1 stabilizes FEN1 and has a pro-viral effect on HCMV lytic replication [18].

The third structural part of hIE1 is formed by an acidic domain (aa 373-475), a highly unordered region [19]. Various groups showed that the SUMOylation site K450 and the interaction site for the signal transducer and activator of transcription (STAT2) are located within this domain [20,21,22]. Paulus et al. revealed that IE1 antagonizes the interferon response by intranuclear relocalisation of STAT2. This results in a reduction of free STAT2 to form the transcription complex IFN stimulated gene factor 3 (ISGF3) together with STAT1 and IFN-regulatory factor 9 (IFN9). Consequently, the reduction of ISGF3 leads to impaired expression of interferon-stimulated genes (ISGs) [23,24]. Interestingly, rat IE1 also contains a highly unordered C-terminal region that could potentially harbour a STAT2 binding domain [14]. Finally, the so-called chromatin tethering domain (CTD) is located at the very C-terminus of hIE1 (aa 476-491). This domain mediates IE1 co-localisation with condensed chromatin and interacts with histones in the chromatin structure [25,26]. Different groups showed that the CTD is not essential for CMV lytic replication. Muecke et al. speculated that the CTD probably plays a role during latency [8,26]. In particular, Tarrant-Elorza et al. reported that a smaller IE1 protein species (IE1x4) is expressed in hematopoietic progenitor cells that are latently infected with HCMV, and this protein appeared to be required for viral genome maintenance [27]. The sequence of the CTD is conserved in primate species but cannot be detected in rat IE1 [8,17,26].

In this study, we investigated whether the similarities in structure between hIE1 and rIE1 can be utilised to characterize the interaction surface of PML. Furthermore, we performed a comparative analysis to identify either species-specific or conserved functions of primate and rodent cytomegalovirus IE1 proteins. Our results demonstrate that while PML binding occurs in a highly species-specific manner, IE1 proteins possess a conserved function in cross-species antagonization of interferon signaling via relocalisation of STAT2.

## 2. Materials and Methods

### 2.1. Oligonucleotide, Plasmids, and Cloning

All oligonucleotide primers used in this study were purchased from Biomers GmbH (Ulm, Germany). All primers and cloned expression plasmids are listed in Table S1. The hIE1_rH mutants were generated using Gibson assembly 1 Step Kit (Synthetic Genomics, La Jolla, CA, USA). Flag-tagged hIE1, hIE1 1-382, and rIE1 expression plasmids [7,14] were used as templates for the polymerase chain reaction (PCR). The helices of rIE1 were generated via PCR using Flag-tagged rIE1 as a template. As the backbone for cloning, Flag hIE1 1-382 was used. Mutants hIE1_rH1, hIE1_rH1/2; hIE1_rH4 and hIE1_rH8 were generated by cloning of one insert via PCR linearised vector. Mutants hIE1_rH2 and hIE1_rH10/11 were generated by cloning three inserts into the linearised Flag pcDNA3 vector [28]. Full-length versions of the hIE1_rH mutants were generated by cleaving the expression plasmids using the fast digest (FD) restriction enzyme XhoI (Thermo Fisher, Waltham, MA, USA) and adding the C-terminus (aa 383-491) of Flag hIE1 via Gibson Assembly. Furthermore, the NanoBRET expression plasmids were purchased from Promega (#N1811). The plasmids were cleaved with the FD enzymes SacI and EcoRI (Thermo Fisher, Waltham, MA, USA). The cloning was also done via Gibson assembly. The hIE1 L174P mutant from Scherer et al. [29], Myc PML [30], and Flag rPML [14] were also inserted in the expression plasmid for the NanoBRET assay. The mutant hIE1 cc172-176 published by Paulus, Harwardt, et al. [31] was also generated. For this, a fragment of hIE1 that carries the cc172-176 mutation was purchased from Integrated DNA Technologies (IDT, Coralville, IA, USA). Then, the segment was integrated via Gibson assembly into Flag IE1 that was cleaved with FD EcoRV (Thermo Fisher, Waltham, MA, USA). Furthermore, besides expression plasmids with Flag-tagged hIE1, hIE1 core, rIE1, and rIE1 core [14], an expression plasmid with a firefly luciferase gene under the control of the UL112 promotor (pHM 142, [32]) and an expression plasmid for IE2 (pHM 134, [33]) were used for the gaussia luciferase assay. Additionally, Schilling et al. generated the Flag-tagged FEN1 176-380 [18] used for cloning the expression plasmids for the NanoBRET assay and the stabilization assay.

### 2.2. Cells and Transfection

HEK293T cells were cultivated in Dulbecco’s minimal essential medium (DMEM) (Gibco, Carlsbad, CA, USA) supplemented with glutamine, 10% fetal calf serum (FCS, Sigma-Aldrich, St. Louis, MO, USA), and 1% penicillin-streptomycin (Sigma-Aldrich, St. Louis, MO, USA). Primary human foreskin fibroblasts (HFF) and rat embryonic fibroblasts (REF), which were obtained from Sebastian Voigt (Berlin, Germany), were maintained in Eagle’s minimal essential medium (MEM, Gibco, Carlsbad, CA, USA) supplemented with 7% FCS, 1% Glutamax (Gibco, Carlsbad, CA, USA), and 1% penicillin-streptomycin (Sigma-Aldrich, St. Louis, MO, USA). HEK293T cells were transfected with 1–2 µg expression plasmid with Turbofect following the manufacturer’s instructions (Thermo Fisher, Waltham, MA, USA). The transfections for the luciferase and NanoBRET interaction assays were done with Lipofectamine 2000 (Invitrogen, Carlsbad, CA, USA) in HEK293T cells.

### 2.3. Stabilization Assay with Cycloheximide Treatment

HEK 293T cells were grown in six well dishes (7 × 10^5^ cells/well). Cells were transfected with expression plasmids for IE1 homologs and human FEN1 aa 176-380 (hFEN1 aa 176-380), followed by the addition of cycloheximide (10 µg/mL, Sigma-Aldrich, St. Louis, MO, USA) at 14 h post-transfection. The cells were harvested after 0, 2, 4, and 6 h of incubation. The resulting lysates were then further analyzed by SDS-PAGE and Western Blotting.

### 2.4. NanoBRET Protein-Protein Interaction Assay

The protein-protein interaction assay was done using the NanoBRET-PPI Kit from Promega (#N1662, Fitchburg, MA, USA). The assay was generally performed following the instructions of the manufacturer. The following steps were changed: 6 × 10^5^ HEK293T cells were seeded in six well dishes. One day later, the cells were transfected with plasmids encoding Halo- and Luc-tagged proteins using Lipofectamine 2000 reagent (Invitrogen, Carlsbad, CA, USA). The DNA ratio of IE1 to human PML and FEN1 aa 176-380 was 1:10, and to rat PML 1:100. After 24 h, the cells were washed once with 1 mL of PBS/EDTA and harvested with trypsin (Sigma-Aldrich, St. Louis, MO, USA). Then, cells were pelleted at 500 rpm for 5 min and resuspended in Opti-MEM™ I reduced serum medium without phenol red (Gibco, Carlsbad, CA, USA). Cell pools of 2.5 mL with 2 × 10^5^ cells/mL were generated. One aliquot of the cells was used for the interaction assay; the other aliquot was used for Western Blot analysis. After the addition of the Halo ligand or DMSO to the pools, the cells were incubated for four h at 37 °C, and the detection of luminescence was done in a plate reader (Hidex Chameleon) for 5 s.

### 2.5. Generation of Lentiviruses and Transduction of HFFs and REFs

HFF and REF cells with doxycycline-inducible expression of human IE1 helix mutants were generated as described elsewhere [14]. The cells were then cultivated in Dulbecco’s minimal essential medium (DMEM, Gibco, Carlsbad, CA, USA)) containing glutamine and supplemented with 10% Tet System Approved FCS (Takara Bio Inc., Shiga, Japan) and 1% penicillin-streptomycin (Sigma-Aldrich, St. Louis, MO, USA).

### 2.6. Indirect Immunofluorescence

HEK293T, HFF, and REF cells were seeded onto coverslips in six well dishes (8 × 10^4^–1.5 × 10^5^ cells/well). The cells were washed three times with 0.01 M PBS after transfection or 24 to 48 h after treatment with doxycycline (Sigma-Aldrich, St. Louis, MO, USA). The cells were fixed in a 4% paraformaldehyde solution (PFA) for 10 min at room temperature (RT). Afterward, the cells were washed four times with 0.01 M phosphate-buffered saline (PBS). The cells were permeabilized through incubation with 0.1% Triton X-100 in PBS for 10 min at 4 °C, followed by a washing step with PBS. After an additional incubation time of 5 min at RT, the respective first antibodies (diluted in PBS with 1% FCS) were added to the cells and incubated for 30 min at 37 °C. Unbound antibodies were removed by washing the cells three times with 0.01 M PBS. Afterwards, the cells were treated with the corresponding fluorescence-coupled secondary antibody diluted in 1% FCS in 0.01 M PBS for 30 min at 37 ˚C. The cells were mounted with DAPI-containing Vectashield mounting medium (Vector Laboratories, Newark, NJ, USA) and analyzed using a Zeiss Axio Observer Z1 with an Apotome2. The pictures were exported using the Zen software. For the quantification of PML foci, Z-series images of 50 cell nuclei per sample were taken, and the number of PML dots was assessed in maximum intensity projection images (0.24 μm distance). Following antibodies were used for immunofluorescence detection: mAb Flag M2 (F1804, Sigma-Aldrich, St. Louis, MO, USA), pAB Flag (F7425, Sigma-Aldrich, St. Louis, MO, USA); mAb PML 5E10 (kindly provided by Roel van Driel) to detect rat PML, and pAB PML A167 + A168 (#A301-167A, #A301-168A, Bethyl Laboratories, Montgomery, AL, USA) to detect human PML. Furthermore, pAB STAT2 from Santa Cruz Biotechnology (#H190, Dallas, TX, USA) was used.

### 2.7. Coimmunoprecipitation

HFF cells expressing human IE1, human IE1 1-382, rat IE1, and rat IE1 1-392 were grown in 10 cm dishes. The expression of the proteins was induced for 48 h with doxycycline (500 ng/mL). Afterwards, the cells were incubated with interferon β (IFNβ; 1000 U/mL; pbl assay science, NJ, USA) for 4 h and then harvested. For this, the cells were washed two times with PBS/EDTA and incubated with Trypsin (Sigma-Aldrich, St. Louis, MO, USA). The cells were harvested with PBS and pelleted at 1500 rpm for 5 min. Next, the cells were lysed in 800 µL lysis buffer (50 mM Tris-HCl [pH 8.0], 150 mM NaCl, 5 mM EDTA, 0.5% NP-40, 1 mM PMSF, 2 μg/mL of aprotinin, 2 μg/mL of leupeptin, and 2 μg/mL of pepstatin) for 30 min on ice. The cells were additionally sonicated for one minute using the QSonica Q700 Sonicator. One part of the lysed cells was used as input control. The other part of the lysates was incubated with anti-Flag antibody M2 (Sigma-Aldrich, St. Louis, MO, USA) coupled to protein-A-sepharose beads (Sigma-Aldrich, St. Louis, MO, USA) for 2 h at 4 °C. The beads were then centrifuged and washed four times with the lysis buffer. After the last step of centrifugation, the buffer was removed, and the beads were boiled in 4× SDS Buffer. The coimmunoprecipitation was then analyzed by SDS-PAGE and Western blotting.

### 2.8. Gaussia Luciferase Assay

HEK293T cells (6 × 10^5^ cells/well) were grown in six well dishes and were transfected with a luciferase reporter plasmid containing the HCMV pUL112/113 promoter together with expression plasmids for IE2 and different IE1 homologs or mutants [32]. The luciferase assay system of Promega (#E1501, Fitchburg, MA, USA) was used. The assay was performed as described by the manufacturer. The measurement was done using the plate reader SpectraMax iD5.

### 2.9. Western Blotting

Lysates from transfected cells were boiled with 4× SDS buffer for 10 min at 95 °C. The lysates were then sonicated for 1 min using the QSonica Q700 Sonicator (QSonica, Newton, MA, USA). Next, the samples were separated on 10% SDS polyacrylamide gels and transferred to PVDF membranes (BioRad, Feldkirchen, Grmany), followed by chemiluminescence detection using a FUSION FX7 imaging system (Vilber Lourmat, Eberhardzell, Germany). Following antibodies were used: mAB Flag M2 (Sigma-Aldrich, St. Louis, MO, USA), pAB human STAT2 H190 (Santa Cruz, Dallas, TX, USA), mAB β-Actin AC15 (Sigma-Aldrich, St. Louis, MO, USA), mAB Halo (G921A, Promega, Fitchburg, MA, USA), pAB IE2 (pAB178; [28]).

### 2.10. RNA Isolation and qPCR

HFF cells expressing human IE1, human IE1 1-382, rat IE1, and rat IE1 1-392 and control cells were grown as triplicates in six well dishes. One day later, the expression of IE1 homologs was induced by adding doxycycline (10 µg/mL). After 24 h incubation, the cells were treated with IFNβ (1000 U/mL) for an additional 24 h. Next, RNA was isolated using the Direct-zol RNA Miniprep Kit (#R2052) from Zymo Research (Irvine, CA, USA) according to the manufacturer’s instructions. Afterward, the Maxima First Strand cDNA synthesis kit (#K1672, Thermo Fisher, Waltham, MA, USA) was used to generate the first strand cDNA. For the reaction, 0.1–0.5 µg RNA was used as a template. The cDNA was diluted 1:4, and 2 µL were used as a template for quantitative real-time PCR (qPCR). Therefore, a master mix was generated that included 10 µL Sso Advanced Univ SYBR Grn. Supermix (BioRad, Feldkirchen, Germany), 7.85 µL nuclease-free water, and 0.15 µL of the following qPCR Primer pair mix: CCL5, CCL8 (Biomol, Hamburg, Germany). Furthermore, the forward and reverse primer of ISG54 were purchased from Biomers (Ulm, Germany). Additionally, the housekeeping gene actin was used for normalisation of PCR values. For this, the primer pair mix for actin (Biomol, Hamburg, Germany) was used. The assay was performed in a 96-well plate, and the Agilent AriaMx Real-time PCR System together with the corresponding software Agilent Aria 1.5 (Agilent Technologies, Santa Clara, CA, USA) was used. The qPCR reaction started with activation of the polymerase for 30 s at 95°C. Afterward, primer binding and strand elongation were done at 60 °C for 30 s for 40 cycles. For the final step, the reaction specificity was verified by a dissociation stage.

### 2.11. Crystal Structure and Bioinformatic Analysis

3D structure predictions of IE1 helix swap mutants were generated using AlphaFold Colab in multimer mode (https://colab.research.google.com/github/deepmind/alphafold/blob/main/notebooks/AlphaFold.ipynb (accessed on 13 June.2022)) [34,35]. As IE1 is known to dimerize [7,14], the models comprising the residues 14-382 of hIE1 were predicted using the multimer mode of AlphaFold; however, only monomers are shown. Structure illustrations were generated with PyMol (PyMOL Molecular Graphics System, Version 1.3. Schrödinger, LLC, New York, NY, USA).

## 3. Results

### 3.1. Establishment of a Robust and Sensitive Interaction Assay for IE1 and PML

**Figure 1:**
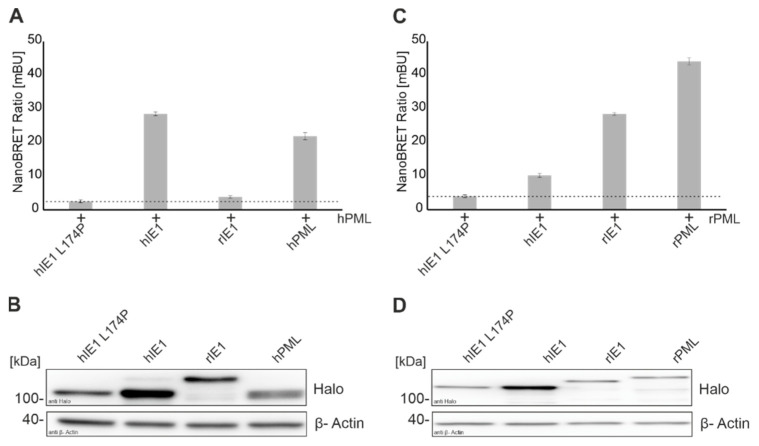
Establishment of a NanoBRET assay for quantitative assessment of IE1-PML interactions. (**A**,**C**) Interaction analysis of IE1 homologs with human PML (**A**) and rat PML (**C**) was performed by transfection of HEK293T cells with Halo tagged IE1 homologs, and Luc tagged PML (Ratios: Halo-IE1: Luc-hPML 1:10; Halo-IE1: Luc-rPML 1:100) for 24 h. After 4 h of incubation with the Halo ligand, emission signals were measured by adding the NanoBRET substrate at wavelengths of 618 nm and 448 nm for 5 s with the Hidex Chameleon. Interactions of IE1 homologs with PML of different species are represented by the mean values ± standard deviation (SD) from three independent experiments with three replicates each. (**B**,**D**) Expression of Halo-tagged proteins was detected in Western blot analyses with a Halo tag antibody and actin as a loading control.

Scherer et al. showed in a previous study that IE1 targets PML via coiled-coil interactions. In contrast to the strong interaction of hIE1 1-382 with PML, only a faint interaction of full-length hIE1 with PML could be detected by coimmunoprecipitation [7]. This suggested that C-terminal sequences of IE1 modulate the interaction with PML. To facilitate the quantitative evaluation of binding between IE1 and PML, we established the NanoBRET protein-protein interaction assay. This method is based on energy transfer caused by the interaction of two proteins from a bioluminescence donor to a fluorescence acceptor [36]. As a readout, the emitted signal of the Halo ligand, which is covalently bound to the Halo tag, is set in relation to the luciferase signal to calculate the NanoBRET ratio, which correlates with interaction strength [36]. This method was established using the well-studied interaction of hIE1 with PML. Firstly, we cloned a NanoLuciferase and a Halo tag on either the N- or C-terminus of PML and IE1. Next, the interaction of all combinations of these proteins was tested, and the highest energy transfer was obtained when PML carried the NanoLuciferase and IE1 the Halo tag at the N-terminus (Figure 1). The IE1 mutant L174P, which does not interact with hPML due to misfolding, was included as a negative control for this assay [29]. Moreover, the dimerization of PML was analyzed and served as a positive control [11]. As shown in Figure 1A, hIE1 and PML yielded a NanoBret ratio which was comparable to the ratio obtained for dimeriziation of PML thus confirming the interaction between these proteins (Figure 1A). Furthermore, Western Blot analyses were carried out to corroborate the correct expression of Halo tagged proteins (Figure 1B). Schweininger et al. recently observed in co-immunoprecipitation experiments that hIE1 does not interact with rPML and vice versa [14]. These observations were also confirmed using this method (Figure 1C,D). To sum up, we successfully established a method for robust and sensitive quantification of the interaction between IE1 and PML proteins.

### 3.2. Generation of Helix Exchange Mutants of hIE1

**Figure 2:**
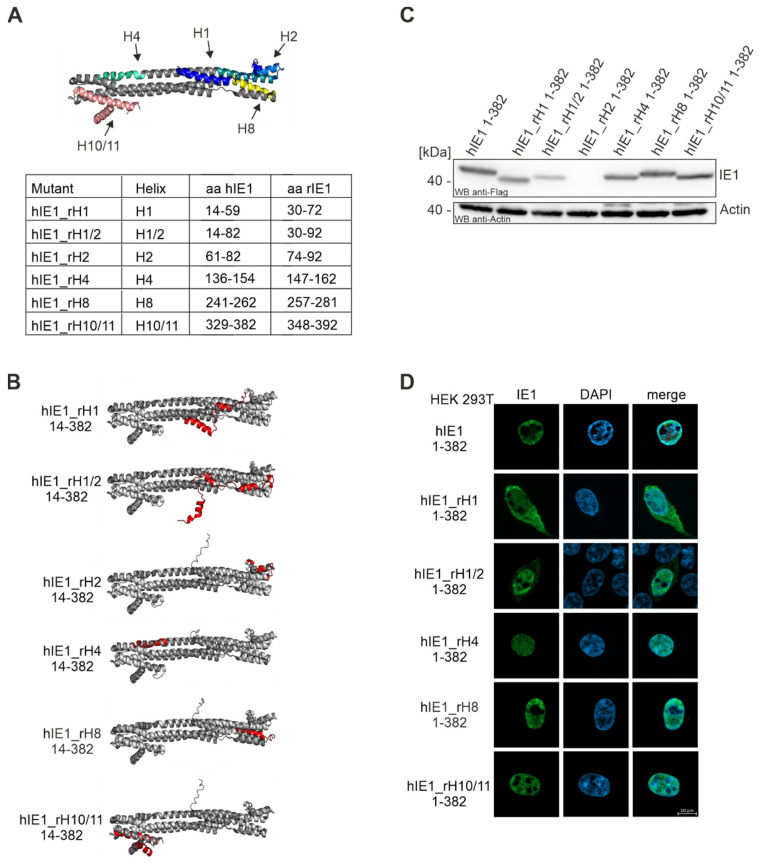
Generation of IE1_rHelix 1-382 mutants by exchanging hIE1 1-382 helices with rIE1 helices. (**A**) Crystal structure of hIE1 14-382 (PDB: 6TGZ, [37], https://www.rcsb.org/ (accessed on 13 June 2022)) with colored helices H1, H1/2, H2, H4, H8, and H10/11 that were swapped with rIE1 helices. The table shows the amino acid region of the respective helices in hIE1 that were exchanged by rIE1 helices. (**B**) Folding predictions of all hIE1_rH mutants using AlphaFold. Models are depicted in dark gray with the exchanged helices colored in red. The crystal structure of hIE1 (PDB: 6TGZ, light gray, [37], https://www.rcsb.org/ (accessed on 13 June 2022)) was superimposed onto the models for reference. (**C**,**D**) Expression analysis of hIE1_rH 1-382 mutants by Western blot and immunofluorescence analysis. HEK293T cells were transfected with expression plasmids for 24 h and harvested for Western Blot or immunofluorescence staining using mAB Flag. A total of 20 cells of each sample were analyzed.

Recently, determination of the crystal structures of hIE1 14-382 and rIE1 30-392 revealed high structural similarity with only small variations, although the sequence identity of the proteins is very low [7,14]. Based on the fold and species-specific interaction of IE1 with PML, we aimed to identify the interaction surface of IE1 with PML. For this, hIE1_ratHelix (hIE1_rH) mutants were generated by swapping hIE1 helices with the corresponding rIE1 helices (Figure 2). Six different rIE1 helices (H1, H1/2, H2, H4, H8, and H10/11) were cloned in the backbone of hIE1 1-382 (Figure 2A). We avoided exchanging helices H6, H7, or H9 since these helices contribute to the dimerization interface of IE1. In the N-terminal head region of hIE1, we exchanged helices 1, 2, 8 and a combination of helices 1 and 2 (H1/2). Helix 4, located in the stalk region of IE1 was also replaced by rIE1 helix 4. At the C-terminus, hIE1_rH10/11 1-382 carries helices 10 and 11 of rIE1. AlphaFold prediction of the respective chimeric proteins suggested that the structural integrity of hIE1_rH mutants is not affected by helix swapping (Figure 2B). Subsequent Western blot analyses of the hIE1_rH mutants revealed that all mutants except hIE1_rH2 were expressed at levels comparable to hIE1 1-382 wild-type (Figure 2C). We assume that swapping of hIE1 helix 2 may have a stronger influence on the structure than predicted by AlphaFold, probably inducing protein misfolding and degradation. Finally, immunofluorescence staining showed nuclear localization of all hIE1_rH 1-382 mutants except for hIE1_rH1 and hIE1_rH1/2 (Figure 2C). These two mutants were not exclusively present within the nucleus but were also found in the cytoplasm. This phenotype is probably caused by a lack of or the weak activity of a nuclear localization sequence in helix 1 of rIE1. In addition to the core versions, we generated the full-length versions of the mutants by adding the C-terminus of hIE1. The full-length versions were expressed as observed for the core versions (data not shown). Taken together, the swap of one or more helices in hIE1 resulted in stable protein expression except for swap mutants for helix 2. The phenotype of these mutants revealed the importance of helix 1 for nuclear localization and helix 2 for hIE1 folding.

### 3.3. Effect of Helix Exchanges in Different hIE1 Regions on PML Binding

**Figure 3:**
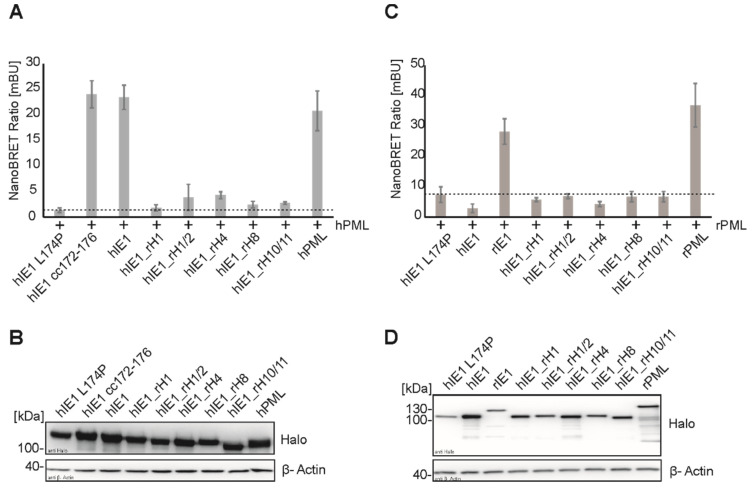
Cross-species interaction of IE1 homologs and IE1_rH mutants with PML by NanoBRET measurements. Interaction of IE1_rH mutants with human PML (**A**) and rat PML (**C**) was done by transfection of HEK293T cells with Halo tagged IE1 versions, and Luc tagged PML for 24 h. After 4 h of incubation with the Halo ligand, the measurement was performed with a detection time of 5 s at wavelengths of 618 nm and 448 nm with the Hidex Chameleon. **(A,B)** Dotted lines indicate the NanoBRET ratio as obtained after coexpression of hIE1 L174P serving as a negative control. (**B**,**D**) The expression of the proteins in lysates of A and C was controlled by Western blotting. The proteins were stained with mAB Halo and mAB actin. Interactions of IE1 homologs with PML homologs are represented by the mean values ± SD from three independent experiments with three replicates each.

To characterize the interaction interface between IE1 and PML, we used the previously established NanoBRET assay to measure the binding of hIE1_rH mutants to PML. As illustrated in Figure 3A,B, all IE1_rH mutants showed a reduced interaction with hPML similar to IE1 L174P. In this experiment, we included the hIE1 cc172-176 mutant as an additional control, as it was recently described by Paulus et al. as a stable protein that no longer binds PML in coimmunoprecipitation analysis [31]. Surprisingly, we measured a strong interaction between hIE1 cc172-176 and hPML in the NanoBRET assay that was comparable to hIE1 and hPML (Figure 3A,B). In addition to the interaction with hPML, we investigated whether the hIE1 mutants carry a rat helix responsible for interaction with rPML (Figure 3C,D). NanoBRET measurements demonstrated that none of the hIE1_rH mutants could interact with rPML. In summary, we found that swapping helices at different positions in hIE1 led to a loss of PML binding and that the integration of rIE1 helices in the hIE1 core did not lead to a gain of interaction with rPML.

### 3.4. Effect of Helix Exchanges in Different hIE1 Regions on PML Disruption

**Figure 4:**
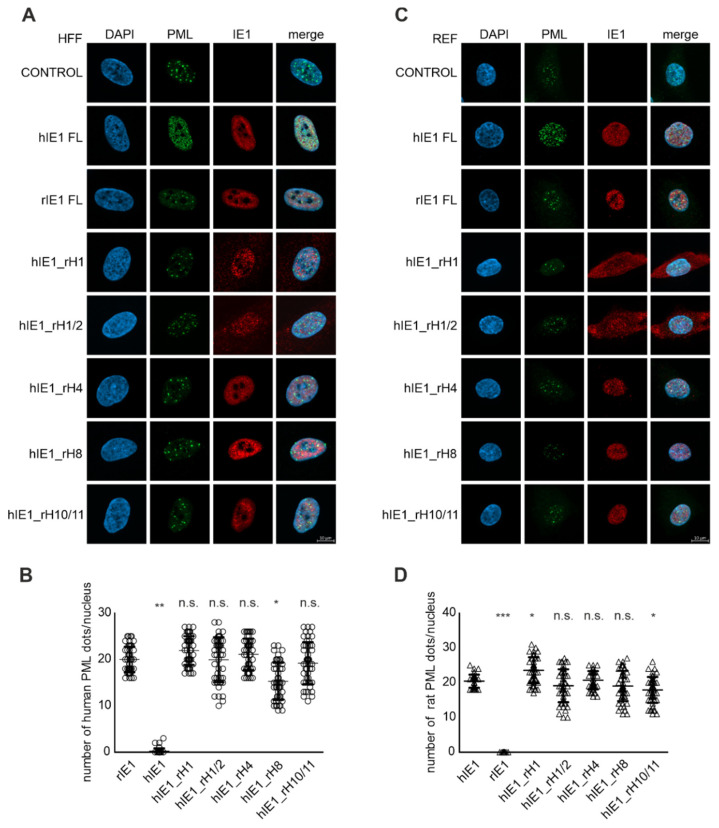
Disruption of PML-NBs by hIE1_rH mutants in inducible cells. Functional analysis of hIE1_rH Mutants in HFFs (**A**,**B**) and REFs (**C**,**D**). The expression of the proteins was induced with doxycycline for 24 h. The cells were fixed for immunofluorescence staining, and the IE1 proteins were detected with mAB Flag. Human PML was stained with pAB A167 and A168 and rPML with mAB 5E10. The number of PML dots in 50 cells from three independent experiments per sample was quantified (**B**,**D**). Statistical significances of PML disruption by IE1_rH mutants were calculated using two-tailed Student’s *t*-test and are indicated by asterisks: * *p* < 0.05; ** *p* < 0.01; *** *p* < 0.001; n.s. = non-significant.

Previous studies showed that IE1 antagonizes the intrinsic immune system by binding to PML, leading to deSUMOylation and dispersion of PML-NBs in the cell nucleus [14,29]. To corroborate the previous interaction results, we investigated the influence of hIE1_rH mutants on PML-NB disruption. For this, cell populations (HFF and REF) that expressed Flag hIE1, Flag rIE1, and Flag hIE1_rH mutants in a doxycycline-inducible manner were generated by lentiviral transduction. Immunofluorescence analyses of HFF cells that were treated with doxycycline revealed that, in contrast to wild-type hIE1, none of the mutants were able to disperse the hPML foci (Figure 4A). Compared to control cells or rIE1 expressing cells, the number of hPML dots (20 dots/nucleus) in cell populations expressing hIE1_rH mutants was not reduced (Figure 4B). A vice versa experiment in REF cells revealed that only wild-type rIE1 was capable of dispersing rPML. However, the hIE1_rH mutants could not disrupt rPML (Figure 4C). Quantifying the number of PML dots in the nucleus confirmed these observations (Figure 4D). Thus, in line with the loss of interaction, replacing hIE1 helices at different places in the structure results in a loss of PML disruption.

### 3.5. Analysis of Flap Endonuclease 1 Binding and Stabilization by hIE1 Helix Exchange Mutants

**Figure 5:**
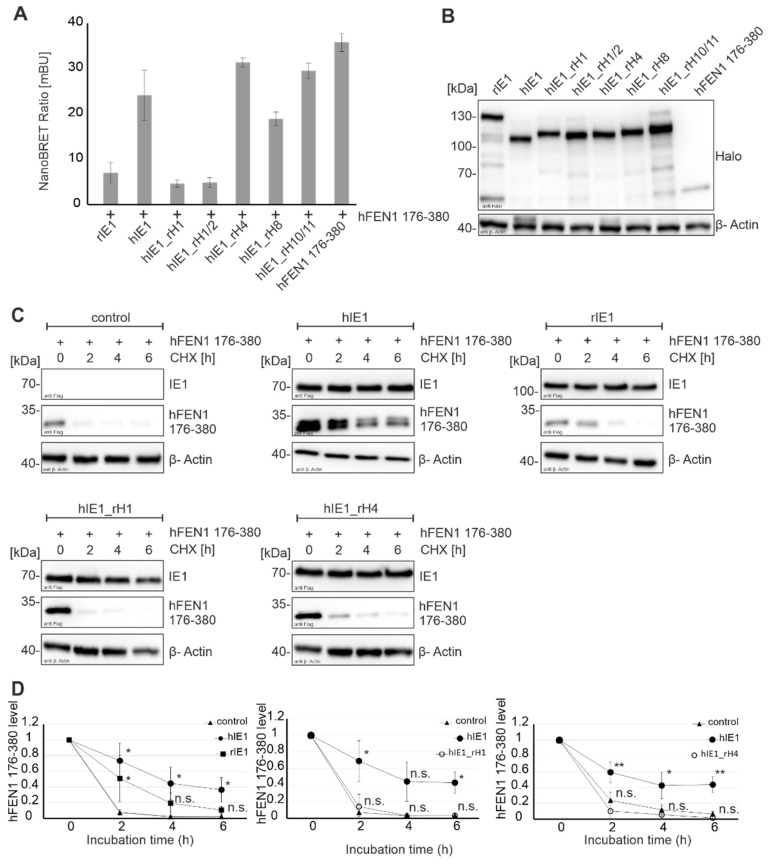
Functional analysis of hIE1_rH mutants with hFEN1. (**A**) NanoBRET measurement of IE1 homologs and hFEN1 was performed. HEK293T cells were cotransfected with Halo tagged IE1 versions, and Luc tagged hFEN1 176-380 for 24 h. Signals of acceptor and donor were measured at 618 nm and 448 nm for 5 s with the Hidex Chameleon plate reader after 4 h incubation with the Halo ligand. (**B**) Protein expression in NanoBRET lysates was controlled via Western blot analysis. The proteins were stained with the mAB Halo antibody and mAB β-actin. (**C**) Stabilization of hFEN1 176-380 by IE1 homologs and hIE1_rH mutants was investigated by cotransfection of HEK239T cells with Flag-tagged IE1 homologs or hIE1_rH mutants and Flag-tagged hFEN1 1 176-380 for 24 h. Cells were treated with cycloheximide (CHX, 10 µg/mL) and harvested after 0, 2, 4, and 6 h of incubation. Western blot analyses were performed to detect Flag hFEN1 176-380, hIE1, hIE1 mutants and β-actin (**D**) Quantification of hFEN1 degradation in independent experiments (rIE1 *n* = 4, hIE1_rH1 and hIE1_rH4 *n* = 3) is represented by mean value ± SD. Statistical significances of hFEN1 176-380 stabilization by IE1 homologs and IE1_rH mutants compared to control were calculated using two-tailed Student’s *t*-test and are represented by asterisks: * *p* < 0.05; ** *p* < 0.01; n.s. = non-significant.

Due to the loss of function of the hIE1_rH mutants in previous experiments, the question arose whether the structural integrity of the respective chimeric proteins was retained. To address this issue, the effect of hIE1_rH mutants on further cellular proteins was examined. Schilling et al. showed that IE1 interacts with human flap endonuclease 1 (hFEN1), stabilising the protein to raise its activity in a pro-viral manner [18]. First, the interaction of rIE1 and IE1_rH mutants with hFEN1 aa 176-380 was analyzed by NanoBRET measurements (Figure 5A,B). While no interaction was detected between rIE1 and hFEN1 aa 176-380, mutants IE1_rH4, IE1_rH8 and IE1_rH10/11 bound to hFEN1 comparable to wildtype hIE1 (Figure 5A). In contrast, mutants hIE1_rH1 and hIE1_rH1/2 lost their ability to interact with hFEN1 aa 176-380. Next, stabilization experiments were performed to investigate whether rIE1 and the mutants could stabilize hFEN1 aa 176-380 (Figure 5C). For this, HEK293T cells were co-transfected with Flag-hFEN1 aa 176-380 together with either Flag-hIE1, rIE1, hIE1_rH1 or hIE1_rH4, respectively. The stability of hFEN1 aa 176-380 was analyzed by inhibiting protein synthesis for different time points using cycloheximide (CHX). As shown in Figure 5C, hFEN1 aa 176-380 was less stabilized by rIE1 than by hIE1 after 2, 4, and 6 h of CHX treatment. Furthermore, Western blot analyses showed that levels of hFEN1 aa 176-380 were reduced in the presence of hIE1_rH1 (Figure 5C, second row, left panel) and hIE1_rH4 (Figure 5C, second row, right panel) to the same extent as the negative control (Figure 5C, first row, left panel). Densitometric analyses of three to four experiments verified these observations (Figure 5D). These experiments demonstrate that while hFEN1 interaction was preserved for individual hIE1 helix swap mutants, hFEN1 stabilization appeared to be species-specific. Interestingly, our results suggest that the interaction of IE1 with hFEN1 is insufficient for FEN1 protein stabilization.

### 3.6. Transactivation of Viral Promotors by Helix Exchange Mutants of hIE1

**Figure 6:**
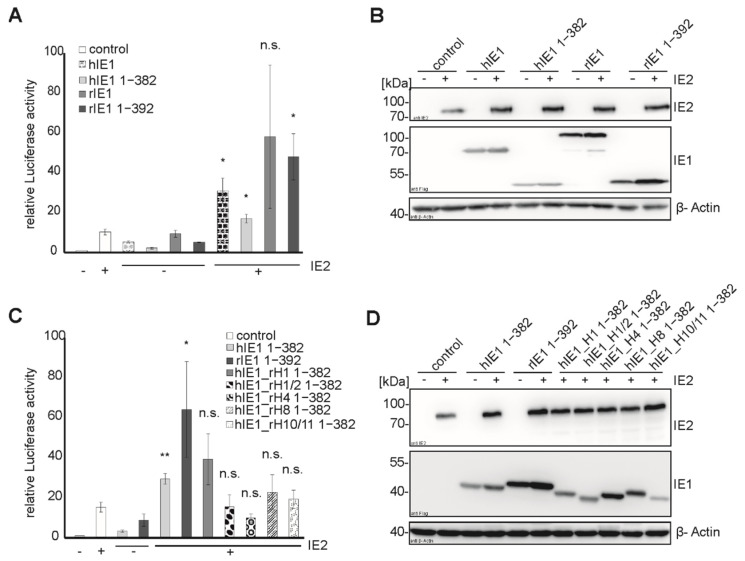
Activation of the HCMV UL112 promoter by rIE1 and hIE1_rH mutants. HEK293T cells were transfected with IE2 and IE1 in different combinations. In all samples, a plasmid encoding the firefly luciferase under control of the HCMV UL112 promoter was cotransfected. 24 h after transfection, the luciferase activity assay was performed. (**A**,**C**) Luciferase assay of IE1 homologs, their core version, and hIE1_rH mutants either alone or in combination with IE2. The transactivation activity of IE1 homologs is represented by mean values ± SD of three independent experiments. Statistical significances of transactivation by IE1 homologs and IE1_rH mutants compared to control were calculated using the Student *t*-test and are indicated by asterisks: * *p* < 0.05; ** *p* < 0.01; n.s. = non-significant. (**B**,**D**) The expression of proteins was controlled by Western blot analyses. The proteins were stained with mAB Flag, pAB IE2, and mAB β-actin.

Regulation of viral and host promoters by IE1 and IE2 is important for lytic replication. IE2 alone has a strong activation effect on viral promoters like the UL112 promotor, but this effect can be enhanced by IE1. Tripathi et al. proposed that the transactivation site of hIE1 is located in the core domain [16]. Further, Sandford et al. showed that rat IE2 could transactivate heterologous promotors, but the activity of rIE1 has not been studied yet [17]. We could confirm weak transactivation of the UL112 promoter by hIE1 and hIE1 core in luciferase assays (Figure 6A, bars 3 and 4). These measurements also demonstrated that rIE1 and rIE1 core could activate this early viral promoter on its own or in combination with IE2 (Figure 6A,B). Next, we analyzed the transactivation activity of the hIE1_rH 1-382 mutants (Figure 6C,D). hIE1_rH1 still showed a transactivation activity like hIE1 WT (Figure 6C, bars 5 and 7). The other four mutants exhibited weak or no activity (Figure 6A, bars 8–11). The luciferase assay revealed that the transactivation of this viral promoter was not species specific. Furthermore, the helix exchanges seem to have different effects on the transactivation function of the hIE1_rH mutants depending on the location of the helices.

### 3.7. Analysis of STAT2 Relocalization by rIE1

**Figure 7:**
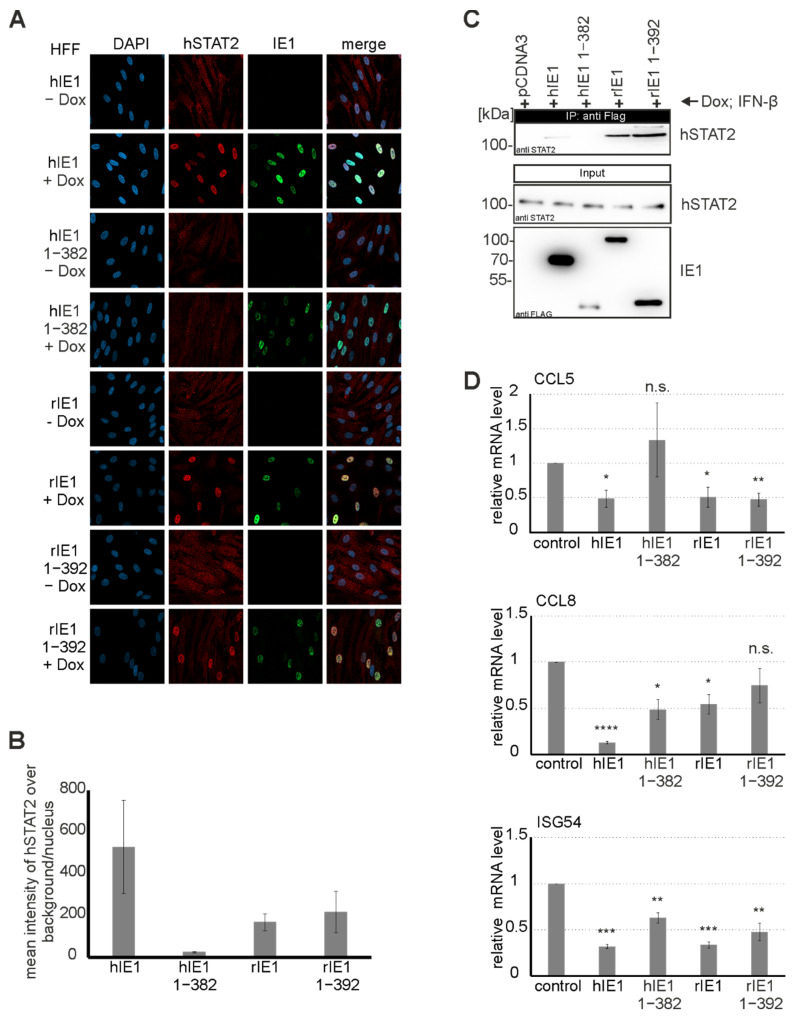
Relocalization and inhibition of STAT2 by rIE1. (**A**) Analysis of STAT2 relocalization by rat IE1 was performed by immunofluorescence staining. The expression of IE1 homologs in HFF cells was induced by adding doxycycline for 24 h. The cells were then fixed, and the proteins were stained with mAB Flag and pAB STAT2 (H190). (**B**) Quantification of relocalization of STAT2 by rIE1 and rIE1 1-382 by measuring the signal intensity of STAT2 in the cell nucleus (50 cells per sample). (**C**) Cross-species interaction of STAT2 with rIE1 and rIE1 1-392 was analyzed by co-immunoprecipitation. The inducible cell lines were treated with doxycycline for 48 h, and the assay was then performed. The IE1 homologs were precipitated using a Flag-specific antibody. Coimmunoprecipitates were analyzed by Western blotting. The proteins were stained with mAB Flag and pAB STAT2. (**D**) Quantification of CCL5, CCL8, and ISG54 expression by qPCR after IFNβ stimulation in the presence or absence of IE1 homologs. Statistical significance of expression levels affected by IE1 proteins compared to control (without IE1) were calculated using the two-tailed Student’s *t*-test and are indicated by asterisks: * *p* < 0.05; ** *p* < 0.01; *** *p* < 0.001; **** *p* < 0.0001; n.s. = non-significant.3.8. Cross-Species Analysis of the Chromatin Tethering Activity of IE1.

Besides counteracting the intrinsic immune system, hIE1 also antagonizes innate immune mechanisms. The highly unordered C-terminus of hIE1 encodes a human STAT2 (hSTAT2) interaction site. Due to the finding that the C-terminal region of rIE1 is also highly unordered, we wanted to investigate whether rIE1 can antagonize hSTAT2 by relocalization as described for hIE1 [14]. First, hSTAT2 relocalization was examined by immunofluorescence staining. For this, expression of IE1 homologs and their core versions was induced in HFF cells by doxycycline. Next, the cells were treated with IFNβ for 4 h. We observed that hSTAT2 was not only relocalized by hIE1 but also by rIE1 (Figure 7A). Interestingly, rIE1 1-392 was sufficient for hSTAT2 relocalization (Figure 7A). Quantification of the intensity of hSTAT2 in the cell nucleus showed a stronger hSTAT2 relocalization by hIE1 than by rIE1 versions (Figure 7B). Coimmunoprecipitation was used to investigate whether hSTAT2 relocalization results from direct interaction with IE1 homologs and their core domains. IE1 homologs expressed in HFF cells were precipitated, and the interaction with endogenous hSTAT2 could be demonstrated (Figure 7C). Due to the relocalization of hSTAT2 by hIE1, the expression of interferon-stimulated genes (ISGs) is reduced [23]. Quantification of mRNA levels by qPCR confirmed the assumption that hSTAT2 relocalization by rIE1 is followed by downregulation of ISG54, CCL8, and CCL5 expression (Figure 7D). To summarize, IE1 from different species can antagonize the innate immune barrier via a similar mechanism. Interestingly, however, the interaction site of hSTAT2 in the IE1 homologs appears to be located at different positions.

### 3.8. Cross Species Analysis of the Chromatin Tethering Activity of IE1

**Figure 8:**
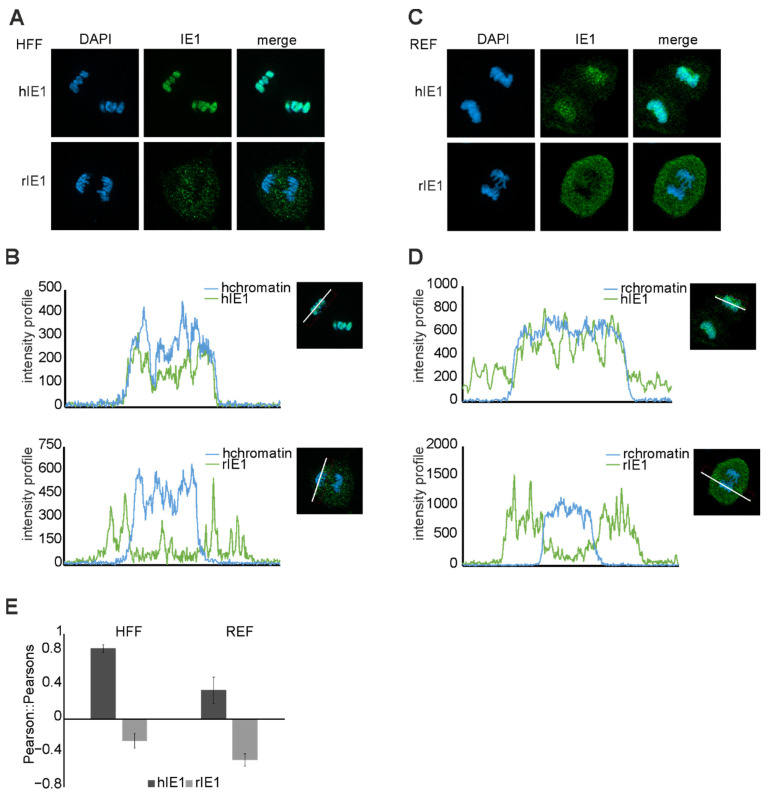
Chromatin tethering by IE1 in cross-species experiments. Immunofluorescence analyses of co-localization of IE1 homologs with mitotic chromatin in HFF (**A**) and REF (**C**) cells. The cells were treated with doxycycline for 24 h and then fixed. IE1 was stained with mAB Flag. Chromatin was stained with DAPI. The intensity profiles (**B**,**D**) and Pearson co-localization coefficients (**E**) were generated by the Zen blue software (30 cell nuclei per sample).

The extreme C-terminus of hIE1 encodes the chromatin tethering domain (CTD) that interacts with histones and remodels chromatin density. While the amino acid sequence of the CTD is conserved in primate species, it is missing in rIE1 [17,26]. The question arose whether rIE1 carries a CTD sequence different from the human CTD sequence. Immunofluorescence analyses of mitotic cells revealed that hIE1 could bind to the chromatin of both species (Figure 8A,C). In contrast, rIE1 was not able to colocalize with chromatin at all. These observations of the immunofluorescence staining were additionally quantified in 30 nuclei per sample using two different methods. First, the intensity profile of the IE1 homologs and DAPI was measured (Figure 8B,D). The second method was the calculation of the Pearson co-localisation coefficient (Figure 8E). Both methods confirmed the observation that only hIE1 can colocalize with chromatin. In line with previous studies, rIE1 does not contain a CTD. Furthermore, hIE1 colocalized with chromatin in a species-independent manner.

## 4. Discussion

IE1 expressed at the beginning of an HCMV infection can counteract innate and intrinsic immune mechanisms. Previous studies demonstrated that PML-NBs form an important intrinsic immune barrier against cytomegaloviruses [10,12,30]. Human IE1 interacts with the main organizer PML, followed by deSUMOylation and disruption of PML NBs. Schweininger et al. showed that the mechanism of antagonizing PML by IE1 seems to be conserved across different species during evolution [14]. Until now, the exact interaction surface of IE1 and PML is unknown. Different groups analyzed the interaction of IE1 with PML using co-immunoprecipitation [7,31]. In the present study, we successfully established a NanoBRET protein-protein interaction assay to measure the interaction of viral proteins with different cellular proteins in a sensitive and quantitative manner (Figure 1). The interaction of IE1 homologs with their natural host PMLs that had already been detected by various groups with distinct methodology could be confirmed [7,14]. In the past years, different hIE1 mutants were generated in an attempt to define the interaction surface of IE1 with PML. NanoBRET analyses verified the observation that the hIE1 L174P mutant no longer interacts with hPML [29]. Surprisingly, however, we detected that hIE1cc172-176, recently described by Paulus et al. as a mutant with wild-type characteristics except for lacking PML binding, still interacted with hPML in a manner comparable to unmodified hIE1 (Figure 3A) [31]. We conclude that clustered charge-to-alanine mutations at positions 172-176 do not abrogate PML binding. Consequently, results obtained with hIE1cc172-176 need to be interpreted with caution [31].

Schweininger et al. solved the crystal structures of the human and rat IE1 core domain [14]. We used the similarities and differences in structure and sequence to search for a potential interaction surface of IE1 with PML. We successfully generated five stable hIE1 core mutants that carry one or two helices of rIE1 (Figure 2). Analyses with AlphaFold prediction suggested that swapping helices should not affect the overall folding of the respective chimeric proteins (Figure 2B). Consistently, five out of six mutants were stably expressed. Only mutant hIE1_rH2 could not be detected in transient expression experiments (Figure 2C). Since helix 2 in rIE1 is displaced by a rotation of 90°, this different angle in hIE1 might cause misfolding of the hIE1_rH2 mutant followed by degradation [14]. Furthermore, immunofluorescence analyses showed that mutants that carry helix 1 of rIE1 were present both in the cytoplasm and nucleus (Figure 2 D). The aa 1-24 in hIE1 function as a nuclear localisation signal [12]. Due to helix exchange, results obtained with mutants hIE1_rH1 and hIE1_rH1/2 revealed that rat helix 1 does either not contain an NLS or the NLS activity contained within rat helix 1 is considerably weaker. This would reinforce the prediction of Sandford et al. for a potential NLS in the C-terminus of rIE1 [17]. NanoBRET measurements demonstrated that the chimeric proteins were no longer able to interact with PML homologs (Figure 3). Moreover, immunofluorescence analyses showed that loss of PML interaction correlated with a defect in PML-NB disruption (Figure 4). Thus, based on these results, we assume that IE1 requires a large surface that comprises more than one helix for successful interaction with PML. This might also explain why the exchange of single amino acids or clustered mutations of IE1 was not able to abrogate PML binding.

In addition to PML, hFEN1 could recently be identified as a binding partner of hIE1 that is hijacked for promoting viral replication [18]. NanoBRET analyses and cycloheximide stabilization assays revealed that rIE1 is neither able to interact with hFEN1 176-380 nor does it mediate hFEN1 protein stabilization (Figure 5A, bar 1). These results indicate that the interaction between hFEN1 and hIE1 is also species-specific. Interestingly, however, we observed that the hIE1 chimeric proteins bearing either helices 4, 8, or 10/11 of rIE1 (hIE1_rH4, hIE1_rH8 and hIE1_rH10/11, respectively) were still able to bind hFEN1 (Figure 5A, bars 5–7). Since mutants that carry helix 1 or helices 1/2 of rIE1 lost their interaction with hFEN1, we assume that the region comprising aa 1-82 of hIE1 may constitute the binding surface for FEN1 or may form an important part of it. In the previous analysis, we detected that mutation of residues located in helices H7 and H9 of hIE1 also interferes with FEN1 binding [18]. One potential explanation for this may be that IE1 dimerization is a prerequisite for hFEN1 interaction, and this may be affected by mutations in helices H7 and H9. Mutants hIE1_rH1 and hIE1_rH4 were then tested for their ability to stabilize hFEN1 176-380. Surprisingly, both mutants exhibited a defect in hFEN1 stabilization while hIE1_rH4 was still able to bind hFEN1. We observed in previous experiments that the binding of hIE1 to hFEN1 not only increases the half-life of the protein but also leads to an accumulation of a hyperphosphorylated hFEN1 isoform [18]. Thus, one may speculate that hIE1 needs to recruit additional cellular factors for hEN1 protein stabilization that interact with C-terminal regions of IE1 affected by the exchange of helix 4.

Concerning the transactivation capacity of the major immediate early proteins of rat CMV, Sandford et al. described that rIE2 could transactivate viral promotors due to similarities in sequence with hIE2 [17]. Despite the low sequence identity of hIE1 and rIE1, we detected a transactivation activity for the core domain of rIE1, as also observed for hIE1, using luciferase assays (Figure 6A,B). Tripathi et al. showed that a possible transactivation site of hIE1 is located in the core domain [16]. Consequently, the integrity of transactivation of the hybrid proteins was tested. Mutant hIE1_rH1 1-382 could transactivate the promoter in the presence of hIE2 like the hIE1 1-382, while hIE1_rH1/2, hIE1_rH4, hIE1_rH8, and hIE1_H10/11 showed either reduced or no activity. The fact that hIE1_rH1 has a strong transactivation capacity suggests that this chimeric protein, which no longer interacts with FEN1, is expressed as a stable and functional protein. Furthermore, this observation supports the assumption that the interaction site of FEN1 is localized within helix 1 of hIE1. In summary, the exchange of helices at different positions of the IE1 core domain affected distinct functions of hIE1. Although all mutants failed to bind PML, other functions were not abrogated by swapping entire helices between hIE1 and rIE1.

Since hIE1 is known to act as a potent interferon antagonist via STAT2 binding at C-terminal sequences, we were interested in investigating whether rIE1 can also modulate STAT2 [23]. Previous studies demonstrated that hIE1 induces an intranuclear relocalisation of STAT2 that abates ISG expression [23,38]. Surprisingly, we observed that full-length rIE1 and rIE1 1-392 lacking the highly unordered C-terminus could induce STAT2 relocalization (Figure 7A). Moreover, rIE1 1-392 exhibited a strong interaction with STAT2 in coimmunoprecipitation experiments indicating that the core domain of rIE1 is sufficient for STAT2 binding (Figure 7B). Interaction with STAT2 correlated with significantly reduced expression levels of selected ISGs upon coexpression of the respective IE1 proteins. This suggests that antagonization of STAT2 by IE1 is conserved and occurs in a species-independent manner. However, primate and rodent CMV IE1 proteins may have evolved distinct binding surfaces to fulfill this task.

Furthermore, hIE1 has been shown to contain a C-terminal chromatin tethering domain (CTD) that plays a role in association with mitotic chromosomes [25,26,39]. Immunofluorescence analyses revealed that rIE1 could neither bind to rats nor human mitotic chromatin (Figure 8). This observation is in line with the observation that rIE1 lacks a sequence similar to the CTD of hIE1 [8,26]. Moreover, our results indicate that hIE1 can colocalize with chromatin of different species. Previous studies demonstrated that the CTD is dispensable for productive replication in asynchronous fibroblast cells [8,25]. However, as suggested by a recent publication using a CTD-deficient recombinant in the Towne strain of HCMV, this domain may be important for maintaining the viral genome during mitosis [39]. If the CTD of hIE1 indeed acts as a genome maintenance factor, one may speculate that either RCMV lacks this function or encodes a different protein that serves to maintain viral genomes during mitosis.

## 5. Conclusions

In summary, we could show that the IE1 proteins of human and rat cytomegalovirus share conserved functions but have also evolved distinctions. While a chromatin tethering activity could only be detected for hIE1, binding to PML, FEN1, and STAT2, as well as transactivation, are conserved between hIE1 and rIE1. However, consistent with the concept of narrow co-speciation, the exact protein surfaces of IE1 for interaction with their respective host factors have diverged during co-evolution.

## Data Availability

Not applicable.

## Supplementary Materials

The following supporting information can be downloaded at: https://www.mdpi.com/article/10.3390/v14081626/s1, Table S1: Sequences of Oligonucleotides.
