# Supplementary material for: Cross-Species Analysis of Innate Immune Antagonism by Cytomegalovirus IE1 Protein
Source: Viruses. 2022 Jul 26;14(8):1626. doi: 10.3390/v14081626 (PMC9331606; doi:10.3390/v14081626)
Supplement: Supplementary file 1 [file viruses-14-01626-s001.zip › viruses-1827512-supplementary.pdf]

Table S1: Sequences of Oligonucleotides

Oligonucleotides for generating hIE1_rH mutants: hIE1_rH1 (PHM 4899), hIE1_rH1/2 (PHM 49889), hIE1_rH4 (PHM 4900); hIE1_rH8 (PHM 4901) and hIE1_rH8 (PHM 4954):
**hIE1_rH1 1-382 (PHM 4899)**
5´rIE1_H1<br>GAGAAAGATGGACCCTGATAATGAACAGCAGCCGGGCGATCG
3´rIE1_H1<br>CAAAAGTTTTGAGGGATTCTTCACTCGGTTCAAAATCGACTTGCACCTC
3´pIE1_1-382_rIE1H1<br>CGATCGCCCGGCTGCTGTTCATTATCAGGGTCCATCTTTCTCTTG
5´pIE1_rIE1 H1<br>GAGGTGCAAGTCGATTTTGAACCGAGTGAAGAATCCCTCAAAACTTTTGAAC
**hIE1_rH1/2 1-382 (PHM 4889)**
3´rIE1_H1/2<br>CCTTAATCTGTTTGACGAGTTCTGCATCGTGGGGAATGAAGTGCTGTTCATC
5´pIE1_rIE1 H1/2<br>GAACAGCACTTCATTCCCCACGATGCAGAACTCGTCAAACAGATTAAG
**hIE1_rH2 1-382 (PHM 4984)**
5´PHM971_MlU1<br>GATGTACGGGCCAGATATACG
3´rIE1_H2<br>GGGGAATGAAGTGCTGTTCATCCATCAGACCTTCCATAGCGACGACTTCCGATTCGGCCAACTCTGGAAAC
3´PHM971_XhoI<br>CTCTAGACTGACTAACTAGATGCATGC
5´pIE1_rIE1 H2^^<br>GTCGTCGCTATGGAAGGTCTGATGGATGAACAGCACTTCATTCCCCACGATGCAGAACTCGTCAAACAGATTAAG
**hIE1_rH4 1-382 (PHM 4900)**
5´rIE1_H4<br>CCTTAGATATCTTAGATAAGCTGATTGCGGCGTCCC
3´rIE1_H4<br>CCTCAGGTACAATGTAGTTCTCCACAATCTTGCCGAGTGCC
5´pIE1_rIE1H4<br>CGGCACTCGGCAAGATTGTGGAGAACTACATTGTACCTGAGGATAAG
3´pIE1_rIE1H4<br>GCCTTGGGACGCCGCAATCAGCTTATCTAAGATATCTAAGGCATTCTGC
**hIE1_rH8 1-382 (PHM 4901)**
5´rIE1_H8<br>GGCACTGCAGAACTTGCCTCAGGTCATCAATGACCAGTCGGTTAGC
3´rIE1_H8<br>GATCAATGTGCGTGAGCACCTTGGCCAGGTTTTCGTTGGCTTCC
5´pIE1_rIE1H8<br>CTGGAAGCCAACGAAAACCTGGCCAAGGTGCTCACGCACATTGATC
3´pIE1_rIE1H8<br>CTAACCGACTGGTCATTGATGACCTGAGGCAAGTTCTGCAGTGC

**hIE1_rH10/11 1-382 (PHM 4954)**
5´rIE1_h10/11<br>GTGTGATGCTGGCCAAGCGGCCTAACGATGCGGTTATTCGCACAC
3´rIE1_H10/11<br>GACACTATAGAATAGGGCCCTCTAGACTGACTAACTAGATGCATGCTCGAGTTAGGACATGCC<br>CACAATCTCTTCC
5´PHM971_MlU1<br>GATGTACGGGCCAGATATACG
3´pIE1_rIE1H10/11<br>GTGTGCGAATAACCGCATCGTTAGGCCGCTTGGCCAGC
Oligonucleotides for cloning of the C-terminus on the hIE1_rH helix mutants: hIE1_rH1 (PHM 4985), hIE1_rH1/2 (PHM 4986), hIE1_rH4 (PHM 4904), hIE1_rH8 (PHM 4987) and hIE1_rH8 (PHM 5016):
5`hIE1_ core_FL<br>GCTCTCCTAGTGTGGATGACCTAC
3´ PHM 971_Xho<br>CTCTAGACTGACTAACTAGATGCATGC
3´hIE1_ rIE110/11_FL<br>GTCGAAAAGCGTGGAAGAGATTGTGGGCATGTCCGCTATTGTAGCCTACACTTTGGC
Oligonucleotides for cloning of hIE1 core, rIE1 core and hIE1_rH mutants (hIE1 1-382 (PHM 4914), rIE1 1-392 (PHM 4917), hIE1_rH1 (PHM 5129), hIE1_rH1/2 (PHM 5130), hIE1_rH4 (PHM 5131), hIE1_rH8 (PHM 5132), hIE1_rH10/11 (PHM 5133)) into eukaryotic expression plasmid pInducer20
5'attB1_FLAG<br>GGGGACAAGTTTGTACAAAAAAGCAGGCTATGGACTACAAAGACGATGA
3'attB2_IE1<br>GGGGACCACTTTGTACAAGAAAGCTGGGTCTTACTGGTCAGCCTTGCTTC
3'attB2_IE1 1-382<br>GGGGACCACTTTGTACAAGAAAGCTGGGTCTTACTCTTCCTCATCTGACTCC
3'rIE1_attB2<br>GGGGACCACTTTGTACAAGAAAGCTGGGTCTCAAGAGCGACGACCACGTTTAG
3'rIE1 1-392_attB2<br>GGGGACCACTTTGTACAAGAAAGCTGGGTCTCAGGACATGCCCACAATCTC
Oligonucleotides for expression plasmids of NanoBRET assay
**Halo tagged plasmids**: hIE1(PHM 4928), rIE1 (PHM 4936), hIE1_rH1 (PHM 5043), hIE1_rH1/2 (PHM 5044), hIE1_rH4 (PH5045), hIE1_rH8 (PHM 5046), hIE1_rH8 (PHM 5047), hIE1 L175P (PHM 5002); hIE1 cc172-176 (PHM 5091), hPML (PHM 4931), rPML (PHM 4940) and hFen1 aa 176-380 (PHM 5029)
5´ N pHT hIE1 FL<br>GATAACGCGATCGCTTCCGAATTCATGGAGTCCTCTGCCAAGAG
3´N pNLF hIE1 FL<br>GATATCCGCGGTTGAGCTCTTACTGGTCAGCCTTGCTTCTAG
5´ C pNLF PML VI<br>CAAAGCGATCGCTTCCGAATTCATGGAGCCTGCACCCGCC
3´C pHT PML VI<br>GAATTGGGCCCAAATCTAGATATCCACAACGCGTTCCTCTCCCTAC
5´N pHT rPML<br>GATAACGCGATCGCTTCCGAATTCATGCCTCCCCCAGAGGAACC

3´ N pNLF rPML<br>CCCAAATCTAGATATCCGCGGTTGAGCTCTTAGGCCAGGCATCCCTTATTTTC
5´ N pHT rIE1 FL<br>GATAACGCGATCGCTTCCGAATTCGATCCGACCCTGTTTACCC
3´N pNLF rIE1 FL rev<br>CTAGATATCCGCGGTTGAGCTCTCAAGAGCGACGACCACG
5´ N pHT Fen1 aa176-380<br>CCACTGAGGATCTGTACTTTCAGAGCGATAACGCGATCGCTATGGCTACCGAGGACATGGACT
3´N pHT_Fen1<br>GGGCCCAAATCTAGATATCCGCGGTTGAGCTCTGATTATTTTCCCCTTTTAAACTTCCC
**Luc tagged plasmids**: hIE1(PHM 4928), rIE1 (PHM 4936), hIE1_rH1 (PHM 5043), hIE1_rH1/2 (PHM 5044), hIE1_rH4 (PH5045), hIE1_rH8 (PHM 5046), hIE1_rH10/11 (PHM 5047), hIE1 L175P (PHM 5002); hIE1 cc172-176 (PHM 5091), hPML (PHM 4931), rPML (PHM 4940) and hFen1 aa 176-380 (PHM 5029)
5´ N PNLF Fen1 aa176-380<br>GCGAACGCATTCTGGCGGGCTCGAGCGGCGCGATCGCTGCTACCGAGGACATGG
3´ N pNLF Fen1<br>GCCCAAATCTAGATATCCGCGGTTGAGCTCTGAATTTATTTTCCCCTTTTAAACTTCCC
5´ C pNLF Fen1 aa176-380<br>GCTTCCGAATTCCTACCGCGGATATCTAGATATGGCTACCGAGGACATGGACT
3´ C PNLF Fen1<br>GAGTGTGAAGACGCCGCTCGAGCCGAGAGCTTTTCCCCTTTTAAACTTCCCTG
5´ N pNLF1 rPML<br>GGGTTCCTCTGGGGGAGGCATGAATTCGGAAGCGATCGCGC
5´N pNLF PML<br>CGGCGCGATCGCTTCCGAATTCATGGAGCCTGCACCCGCC
3´N pNLF PML<br>CCCAAATCTAGATATCCTTACCACAACGCGTTCCTCTCCC
Oligonucleotides for the cloning hIE1 cc172-176 mutant of Paulus *et al.*
3´cca172-176 IE1<br>CATTGTTTCCACACATGTAGTGAGG
5´cca172-176 IE1<br>GAGGATGTTTGCAGAATGCCTTA
Oligonucleotides for RT-qPCR
5`ISG54<br>ATGTGCAACCTACTGGCCTAT
3´ISG54<br>TGAGAGTCGGCCCATGTGATA
